# Association Between the Growth of Accountable Care Organizations and Physician Work Hours and Self-employment

**DOI:** 10.1001/jamanetworkopen.2018.0876

**Published:** 2018-07-27

**Authors:** Anwita Mahajan, Lucy Skinner, David I. Auerbach, Peter I. Buerhaus, Douglas O. Staiger

**Affiliations:** 1 Department of Economics, Massachusetts Institute of Technology, Cambridge; 2 Center for Interdisciplinary Health Workforce Studies, Montana State University College of Nursing, Bozeman; 3 Department of Economics, Dartmouth College, Hanover, New Hampshire; 4 National Bureau of Economic Research, Cambridge, Massachusetts

## Abstract

**Importance:**

The share of the population covered by accountable care organizations (ACOs) is growing, but the association between this increase and physician employment is unknown.

**Objective:**

To investigate the association between the growth of ACOs and changes in physician work hours, probability of being self-employed, and probability of working in a hospital.

**Design, Setting, and Participants:**

A fixed-effects design was used in this cross-sectional study to compare changes in physician employment in hospital referral regions with high vs low ACO growth. A nationally representative 1% sample of all working US physicians obtained annually from 2011 through 2015 from the American Community Survey (N = 49 582) was included. Data analysis was conducted from March 28, 2017, to April 10, 2018.

**Main Outcomes and Measures:**

Physician hours worked per week, probability of being self-employed, and probability of working in a hospital.

**Results:**

Of the 49 582 physicians included in the study, 63.5% were men; the mean (SD) age of sampled physicians was 46.01 (11.59) years. In 2011, sampled physicians worked a mean (SD) of 52.2 (16.1) hours per week, 24.43% were self-employed, and 42.03% worked in a hospital. A 10–percentage point increase in ACO enrollment in a hospital referral region was associated with a statistically significant reduction of 0.82 (95% CI, −1.52 to −0.13; *P* = .02) work hours in men and a decrease of 2% (95% CI, −3.8% to −0.1%; *P* = .04) in the probability of all physicians being self-employed. The association with self-employment was strongest (−5.0%; 95% CI, −8.7% to −1.4%; *P* = .006) in physicians aged 50 to 69 years, who were also more likely (4.0%; 95% CI, 1.0% to 6.9%; *P* = .009) to work in a hospital.

**Conclusions and Relevance:**

The growth of ACOs within hospital referral regions appears to be associated with a reduction in hours of work and self-employment among physicians. These results suggest that ACOs may affect physician employment patterns.

## Key Points

**Question:**

Is the growth of accountable care organizations associated with changes in physician work hours, probability of being self-employed, and probability of working in a hospital?

**Findings:**

In this cross-sectional study including 49 582 physicians, a 10–percentage point increase in accountable care organization enrollment in a hospital referral region was associated with a statistically significant reduction of 0.82 work hours per week among male physicians. In addition, the 10–percentage point increase was associated with a decrease of 2% in the probability of all physicians being self-employed.

**Meaning:**

These results suggest that accountable care organizations may affect physician employment patterns.

## Teaser

This cross-sectional study evaluates the association between the expansion of accountable care organizations throughout the United States and changes in physician work hours, self-employment, and employment in hospitals.

## Introduction

Accountable care organizations (ACOs) consist of physicians, hospitals, and other professionals who are held accountable for the cost and quality of care received by covered patients.^1^ Fueled by the passage of national health reform in 2010, ACOs have grown rapidly, with 923 ACO contracts in 2017 covering more than 32 million people in the United States.^2^ The limited number of studies on ACOs indicate that they have generated small savings and some improvements in quality.^3,4^

Despite their rapid growth, the associations between ACOs and physician work hours and self-employment are unknown. In 2010 Kocher and Sahni^5^ suggested that ACOs’ acquisition of medical practices and employment of physicians could reduce incentives for physicians to work long hours and that hospital-controlled contracts could result in a decrease in physician self-employment. However, we are unaware of empirical evidence investigating these possible effects on physician employment patterns.

In this study, we examine whether the growth of ACOs is associated with changes in physician work hours and self-employment, including being employed by a hospital. The growth of ACOs has occurred unevenly across the country, with some hospital referral regions (HRRs) now having more than 20% of their population covered by ACOs and others having close to no ACO coverage (Figure). We compared physician work hours and self-employment between HRRs that have had more and less rapid growth of ACOs.

**Figure.**
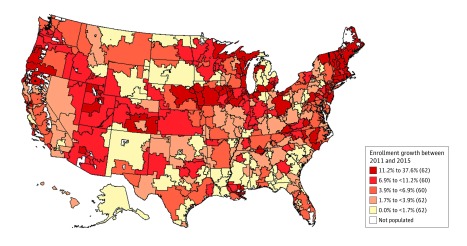
Change in Accountable Care Organization (ACO) Enrollment in Hospital Referral Regions (HRRs) Geographic distribution in ACO enrollment growth across HRRs in the United States. ACO enrollment was defined as the percentage of an HRR’s population covered under an ACO contract (D. Muhlestein, PhD, JD, of Leavitt Partners, written communication, June 24, 2016). Parenthetical values indicate the number of HRRs with ACO enrollment growth in each range.

## Methods

Data on physicians came from the American Community Survey (ACS) for 2011-2015, which is a 1% sample of the US population conducted annually by the US Census Bureau.^6^ The ACS is a nationally representative survey with a response rate of more than 95%. It is illustrative of all physicians living and working in the United States and has been shown to more accurately represent the physician workforce than other widely used physician surveys.^7^

To avoid potential selection bias if certain types of physicians are more likely to work in ACOs, our sample included all working physicians (N = 49 582) regardless of whether they are associated with an ACO. Physicians were associated with an HRR by mapping the public-use micro area of their residence available in the ACS to HRRs, using MABLE/Geocorr, version 14 (Missouri Census Data Center). The ACO enrollment rates from 2011 to 2015 were defined as the percentage of an HRR’s population covered under an ACO contract (D. Muhlestein, PhD, JD, of Leavitt Partners, written communication, June 24, 2016).

Individuals in the ACS were identified as working physicians if they reported working for pay in the prior week and described their occupation as physician or surgeon. Physician weekly hours worked were self-reported based on the number of hours they usually worked each week (in weeks that they worked) over the prior 12 months. In addition to basic demographic information, these respondents were asked the industry of their employer (which we used to determine whether they worked for a hospital) and whether they were self-employed (vs an employee).

The ACS is public use data that do not contain identifiable information, and the ACO enrollment data are available only at the HRR level where individuals are not identifiable. Therefore, the Dartmouth College Institutional Review Board stated that this study was exempt from institutional review board review. This study followed the Strengthening the Reporting of Observational Studies in Epidemiology (STROBE) reporting guideline.

### Statistical Analysis

A fixed-effects model was used to examine within-HRR variation in ACO enrollment growth between 2011 and 2015. This model controls for fixed differences across HRRs and compares changes in employment characteristics for physicians in HRRs with rapid ACO enrollment growth with physicians in HRRs with little or no enrollment growth. More specifically, we estimated linear regressions using 3 different dependent variables: physician weekly hours worked, a continuous variable; hospital employment, a dummy variable that took the value 1 if the physician reported being employed by a hospital and 0 if otherwise; and self-employment, a dummy variable that took the value 1 if the physician reported being self-employed and 0 if an employee was working for salary or wages. The key independent variable was the ACO enrollment rate in each physician’s HRR, defined as the percentage of total lives in an HRR covered under an ACO contract.^8^ Regressions included year dummies to control for time trends, HRR indicators to control for fixed differences across HRRs, and variables to control for physician citizenship status, marital status, family size, race/ethnicity, sex, and age. Physicians younger than 25 years and older than 69 years were excluded. By controlling for HRR indicators (the HRR fixed effects), the regressions control for fixed differences across HRRs and estimate the association between changes over time in ACO penetration and changes over time in physician employment characteristics within an HRR.

Statistical significance was tested using a 2-sided *t* test of whether the coefficient on ACO penetration was equal to 0. Statistical significance is reported at the .10, .05, and .01 levels. Tests of significance accounted for the use of sampling weights and for clustering of observations within an HRR. Analyses were performed using Stata, version 14 (StataCorp).

## Results

Accountable care organization enrollment growth varied considerably, with HRRs in the top quintile of ACO growth experiencing between 11– and 38–percentage point growth in ACO enrollment between 2011 and 2015; HRRs in the bottom quintile experienced 0– to 1.7–percentage point growth (Figure). Hospital referral regions with high and low ACO enrollment growth were distributed evenly across the country. The mean (SD) ACO enrollment across all HRRs was 1.14% (1.87%) in 2011, which grew to 9.13% (6.75%) in 2015 (Table 1).

**Table 1.** Summary of Variables^a^

Variable^b^	Year 2011 (n = 8274)	Year 2015 (n = 8572)
ACO enrollment (% of health care market), mean (SD)	1.14 (1.87)	9.13 (6.75)
Physician outcomes		
Hours worked, mean (SD)	52.2 (16.1)	51.87 (15.70)
Self-employed, %	24.43	20.28
Work in hospital, %	42.03	46.57
Physician characteristics		
Men, %	63.29	63.08
Young (25-49 y), mean %	60.29	60.59
Age, mean (SD), y	45.97 (11.31)	45.91 (11.83)
Married with spouse present, %^c^	75.91	74.25
White race, %^d^	72.33	72.49
US citizen, %^e^	71.11	71.29
Family size of ≤3, %	63.79	65.29

Abbreviations: ACO, accountable care organization.

^a^ Estimates are from data on all employed physicians from 2011 to 2015 (N = 49 582) from the American Community Survey.^6^ All summary estimates are weighted by sampling weights provided by the American Community Survey.

^b^ For the categorical variables, means are the percentage of the population in the given category.

^c^ Physicians who did not report being married with spouse present reported being married with spouse absent, separated, divorced, widowed, or single.

^d^ Physicians who did not report their race as white reported being black, American Indian or Alaska Native, Chinese, Japanese, other, of 2 major races, or of 3 major races.

^e^ Physicians who did not report being US citizens reported being born abroad of American parents, naturalized citizens, or not citizens.

In 2011, physicians worked a mean (SD) of 52.2 (16.1) hours per week, with 24.43% self-employed and 42.03% working in a hospital (Table 1). By 2015, mean (SD) hours worked decreased slightly to 51.9 (15.7), self-employment fell to 20.3%, and the percentage of physicians working in hospitals increased to 46.6%. Overall, 63.5% of the physicians were men and 60.0% were younger than 50 years (mean [SD] age of sampled physicians, 46.01 [11.59] years).

Table 2 reports the results of multiple regression analysis that estimated the association between physician employment variables and a 10–percentage point increase in ACO enrollment in the HRR in which the physician lived. A 10–percentage point increase in ACO enrollment was associated with a decrease of 0.62 work hour per week (95% CI, −1.31 to 0.07 hours; *P* = .08). Although ACO penetration was associated with fewer hours worked for both women and men, only the association for men was significant (0.82 hour; 95% CI, −1.52 to −0.13 hours; *P* = .02). For young physicians (age, 25-49 years), a 10–percentage point increase in ACO enrollment was associated with a decrease of 0.86 hour worked per week (95% CI, −1.74 to 0.02 hours; *P* = .06). There was no significant association in hours worked among older physicians (age, 50-69 years) (−0.05 hour; 95% CI, −1.07 to 0.98 hours; *P* = .09).

**Table 2.** Association of an Increase in Accountable Care Organization Penetration From 0% to 10% With Physician Weekly Hours Worked, Probability of Self-employment, and Probability of Working in a Hospital^a^

Physician Subset	Weekly Hours Worked, h (95% CI)	*P* Value	Self-employed, % (95% CI)	*P *Value	Work in Hospital, % (95% CI)	*P *Value
All physicians	−0.62 (−1.31 to 0.07)	.08	−2.0 (−3.8 to −0.1)	.04	1.6 (−0.7 to 3.9)	.17
Men	−0.82 (−1.52 to −0.13)	.02	−2.1 (−4.3 to 0.2)	.08	1.8 (−0.7 to 4.2)	.16
Women	−0.27 (−1.42 to 0.89)	.65	1.6 (−3.8 to 0.5)	.14	0.7 (−3.1 to 4.6)	.71
Age, y						
25-49	−0.86 (−1.74 to 0.02)	.06	−0.1 (−2.2 to 1.9)	.90	0.2 (−2.9 to 3.4)	.89
50-69	−0.05 (−1.07 to 0.98)	.93	−5.0 (−8.7 to −1.4)	.006	4.0 (1.0 to 6.9)	.009

^a^ Estimates are from regressions using data from the American Community Survey on all employed physicians from 2011 to 2015 (N = 49 582).^6^ All regressions controlled for physician age, family size, and indicators for year, hospital referral region, citizenship status, marital status, race, and sex. All regressions were weighted by sampling weights provided by the American Community Survey, and *P* values and 95% CIs accounted for the use of sampling weights and for clustering within hospital referral regions.

Table 2 also indicates that a 10–percentage point increase in ACO enrollment in the HRR in which the physician lived was associated with a decrease in the probability of being self-employed (−2.0%; 95% CI, −3.8% to −0.1%; *P* = .04), a result particularly apparent among older physicians. Although the association with being self-employed was not significant among women (*P* = .14), it was significant among men (2.1%; 95% CI −4.3% to 0.2%; *P* = .08). Results also indicate a 5.0% decreased probability of being self-employed (95% CI, −8.7% to −1.4%; *P* = .006) and a 4.0% increased probability of being employed in a hospital (95% CI, 1.0% to 6.9%; *P* = .009) among older physicians.

## Discussion

In 2010, Kocher and Sahni^5^ suggested that hospital-controlled ACOs would acquire practices and hire physicians as employees, which would reduce the physicians’ incentive to work long hours. This implication seems to be borne out in our results that indicate a reduction in work hours and self-employment associated with growth in ACOs. Although our data do not offer a mechanism that underlies these findings, well-performing ACOs may make more efficient use of physician time by shifting tasks that do not require physicians to other team members.^9^ The decreases in hours worked were not large in our results; however, ACOs are still relatively new and, as they expand and become more administratively mature, physician hours could decrease further. Furthermore, although an overall trend of increased physician employment in hospitals was observed in 2015,^10^ our results suggest the possibility that these trends may be linked to high ACO growth. The role of ACOs in decreasing self-employment is anecdotally corroborated by the observations of physicians opting into hospital-controlled ACOs for the security they afford.^11^

### Limitations

Our data did not allow for identification of whether each physician worked in an ACO because we relied on an estimate of the ACO penetration in the HRR in which the physician lived. However, ACO growth may lead to market-level changes that affect hours and employment choices of all physicians in an HRR—not just those who directly work in an ACO. For example, ACO growth in a market may put downward pressure on reimbursement for all physicians, and lower physician fees have been found to be associated with declines in physician hours.^12^ Similarly, recent data suggest that new physicians entering the labor market are more likely to work in large groups, irrespective of whether they are employed in an ACO.^13^ Thus, the large decline in self-employment that we found to be associated with the growth of ACOs is likely to reflect how ACOs are affecting a population of physicians beyond that directly employed by ACOs. Another limitation of the ACS data is that physician specialty is not identifiable. Therefore, we could not explore whether the growth of ACOs was more associated with changes in hours and employment choices for particular specialties, such as primary care. As with any observational study, the association of physician hours and employment choices with ACO growth may not be causal. In particular, the growth of ACOs might be a proxy for broader market-level reforms that are affecting these HRRs. Furthermore, the direction of causation between ACOs and physician employment could be the reverse of what we identified: ACOs could be arising as a response to changing employment preferences in physicians. This seems unlikely, however, given the rapid rise of ACOs following their creation as part of the Affordable Care Act.

## Conclusions

Future research should investigate whether and how ACOs and other organizational structures affect physician employment outcomes and work satisfaction. The strongest association between ACO growth and self-employment in this study was seen in older physicians. It is possible that older physicians are shifting to employment and selling their practices to hospitals to receive retirement benefits.^14^ Such retirement could fuel further increases in ACO employment in the short run, but in the long run could raise questions about availability of physicians in ACOs. Given the wave of concern over physician burnout, it will be important to assess job satisfaction among physicians in ACOs to understand whether ACOs might help to alleviate some of the concerns currently besetting physicians. Given the rapid increases in the numbers of primary care nurse practitioners, future research should also examine the association between ACOs and employment patterns of these nonphysician clinicians.
